# Ruminal archaea and bacteria metatranscriptomic responses to supplementation in steers fed low-quality forage

**DOI:** 10.1093/jas/skag188

**Published:** 2026-06-12

**Authors:** Alyson B Fontenot, Laura Z Holland, Jodi R Cox, Taylor A Falk, Sapna Chitlapilly-Dass, Matthew R Dunn, Brigid R Arciero, Lauren E Fitch, Tryon A Wickersham

**Affiliations:** Department of Animal Science, Texas A&M University, College Station, TX, 77843, United States; Arkea Bio Corp, Charlestown, MA, 02129, United States; Department of Animal Science, Texas A&M University, College Station, TX, 77843, United States; Arkea Bio Corp, Charlestown, MA, 02129, United States; Department of Animal Science, Texas A&M University, College Station, TX, 77843, United States; Arkea Bio Corp, Charlestown, MA, 02129, United States; Arkea Bio Corp, Charlestown, MA, 02129, United States; Arkea Bio Corp, Charlestown, MA, 02129, United States; Department of Animal Science, Texas A&M University, College Station, TX, 77843, United States

**Keywords:** beef cattle, rumen, low-quality forage, supplementation, archaea, metatranscriptomics

## Abstract

A basal diet of low-quality forage (LQF) results in decreased forage utilization and animal performance. Protein and starch supplementation can ameliorate these effects by providing additional nitrogen and energy to ruminal microbes. However, the effect of protein and starch supplementation on ruminal microbial gene expression, specifically pathways involved in methanogenesis, is not well-documented. We aimed to evaluate the effect of supplement composition on ruminal microbial gene expression before and after supplementation in steers consuming a basal diet of LQF. Five ruminally-cannulated Angus steers (BW 375 ± 45 kg) were used in a 4 × 4 Latin square and fed a basal diet of King Ranch Bluestem Hay (3.5% CP, 73% NDF), and one of four isonitrogenous (130 mg nitrogen/kg BW) supplements. Ruminal samples collected from the steers provided the two most divergent supplements, 2% starch (43% CP; 100% soybean meal) and 56% starch (21% CP; 78% corn, 19% soybean meal, 2.4% urea) were used for metatranscriptomic analysis. Samples were collected via cannula prior to and 4 h post-supplementation; total RNA was sequenced. Differentially expressed genes (DEG) were identified and functionally annotated; pathway enrichment analysis was completed. Sampling time exerted a greater effect than starch concentration (∼78 vs. 17% in both archaea and bacteria). All methanogenesis pathways were differentially regulated by time (*P *< 0.05) with upregulated genes primarily expressed by Thermoplasmata and downregulated genes primarily expressed by *Methanobrevibacter.* Acetoclastic and methylotrophic methanogenesis pathways were differentially regulated by starch supplementation (*P *< 0.05) with upregulated genes primarily expressed by Thermoplasmatales and downregulated genes primarily expressed by *Methanomethylophilus alvus.* The majority of bacterial DEG were expressed by Clostridia, but unclassified Bacteroidetes was the predominant bacteria responsible for expression of carbohydrate active enzymes (CAZymes). Time relative to feeding, rather than starch supplementation, resulted in a greater number of bacterial CAZymes expressed. Supplement composition elicited changes in the taxa involved in methanogenesis and carbohydrate metabolism, suggesting the potential for effects on CH_4_ emissions. Further research is needed to directly quantify CH_4_ emissions in response to supplementation to potentially optimize supplementation strategies and improve sustainability of beef cattle production.

## Teaser

Time post-feeding, rather than concentration of starch supplementation, had a greater effect on ruminal archaeal and bacterial gene expression in steers consuming low-quality forage and supplemental protein.

## Introduction

Ruminants rely on microbial fermentation to digest human inedible, fibrous plant material to meet their energy and protein requirements. To accomplish this, ruminal bacteria utilize a complex network of carbohydrate active enzymes (CAZymes) for organic matter degradation. The majority of CAZymes responsible for fiber digestion in the rumen belong to the glycoside hydrolase (GH) family, a class of enzymes that hydrolyze glycosidic bonds between carbohydrates or carbohydrates and a non-carbohydrate molecule (Krause et al. 2003). Other CAZyme families include glycoside transferases (GT; form glycosidic bonds), carbohydrate esterases (CE; remove ester groups from carbohydrates), polysaccharide lyases (PL; cleave uronic acid-containing chains), and carbohydrate binding modules (CBM; substrate binding modules for GH; Nakamura et al. 2017; Chettri et al. 2020; Sidar et al. 2020). Bacteria utilize CAZymes to degrade complex polysaccharides into their respective monosaccharides which are subsequently fermented into volatile fatty acids (VFA), generating H_2_ and CO_2_ which serve as substrates for methanogenic archaea (Elsden 1945; Bergman 1990; Johnson and Johnson 1995). Carbohydrate fermentation is the primary source of VFA and CH_4_ production. Therefore, it is critical to evaluate both bacterial and archaeal activity to fully understand methanogenic pathways.

Methanogenesis occurs via hydrogenotrophic, methylotrophic, or acetoclastic pathways, with hydrogenotrophy being the predominant pathway in the rumen (Morgavi et al. 2010). The final reactions of methanogenesis are common to all methanogenesis pathways, regardless of substrate (Ferry 2011). Briefly, the methyl group in methyl-Coenzyme M (HS-CoM) is reduced by methyl-coenzyme M reductase (*Mcr*), using Coenzyme B (CoB) as an electron donor, resulting in CH_4_ and a CoM-CoB heterodisulfide bond. Heterodisulfide reductase (*Hdr*) is necessary to cleave the CoM-CoB heterodisulfide bond and regenerate CoM and CoB for methanogenesis to continue. Formate dehydrogenase (*fdh*) and F420 non-reducing hydrogenase (*mvh*) are coupled with specific subunits of *hdr.* According to the KEGG database, genes coding for *mcr, hdr, fdh*, and *mvh* are involved in all methanogenic pathways. However, some authors have observed differences in *hdr* subunits and the *hdr-mvh-fdh* complex between methanogenic taxa (Hedderich et al. 1989; Buan and Metcalf 2010; Refai et al. 2014; Watanabe et al. 2021). Interpretation of results in this paper will use the enriched pathways identified by the KEGG database.

Efficient ruminal microbial fermentation is critical for cattle to meet their energy and protein requirements. Prolonged energy and protein deficiency, caused by a basal diet of low-quality forage (LQF; crude protein < 7%), results in decreased forage organic matter intake (FOMI) and digestion, subsequently reducing animal performance (Cochran et al. 1998). As forages mature, protein concentration decreases and cell-wall components (cellulose, hemicellulose, and lignin) increase, decreasing both intake and digestion (Buxton 1996). Decreased FOMI and digestion is a consequence of deficient ruminally available nitrogen inhibiting bacterial growth and activity, resulting in reduced VFA and microbial crude protein (MCP) production (Asplund et al. 1958; Cochran et al. 1998). Supplementation with protein ameliorates some negative effects of feeding LQF (Lee et al. 1985; McCollum and Galyean 1985; Olson et al. 1999). Supplementing starch in combination with protein can further increase total digestible organic matter intake resulting in greater VFA concentrations and improved animal performance (Heldt et al. 1999). While the effect of supplementation on intake and digestion is well-documented, little is known about the microbiome response. Supplementation to cattle consuming LQF has been observed to alter ruminal bacterial populations (Latham et al. 2018; McCann et al. 2014); however, to the best of our knowledge, the effect of supplementation on ruminal prokaryotic gene expression has not been evaluated. Therefore, the objective of this research was to evaluate the effect of supplement composition on expression of ruminal archaeal genes involved in methanogenesis and bacterial genes involved in carbohydrate degradation and metabolism in steers consuming LQF.

## Materials and methods

### Animals, diets, and ruminal fluid collection

Animal care and experimental procedures were conducted according to the guidelines of Texas A&M University Institutional Animal Care and Use Committee #2022-0223. Five *Bos taurus taurus* (Angus) ruminally-cannulated steers housed in a climate-controlled metabolism barn were used in a 4 × 4 Latin square, with an additional column for steer, to determine the effect of protein and starch supplementation on forage utilization, nitrogen balance, and urea kinetics (Cox and Wickersham 2023, 2024). All steers had *ad libitum* access to a basal diet of LQF determined as 130% of the previous five-day average. Hay provided was chopped (76 × 76 mm wire mesh screen) King Ranch Bluestem (3.5% CP). The four treatments were 2, 20, 38, and 56% starch. All supplements were fed to provide 130 mg of nitrogen/kg BW and consisted of commercially available feedstuffs (Table 1). Formulating supplements with conventional feedstuffs creates the potential for confounding the results while also increasing the utility of the results. Concentration of nitrogen and starch supplementation was selected based on previous research from our lab and supplements were formulated to closely mimic supplements commonly fed in the southern plains. The 2% and 56% starch supplements were designed to mimic the protein and energy content of commercially available 40 and 20% CP range cubes. Due to the sequencing and computational costs associated with metatranscriptomics, the two most divergent treatments 2% starch (100% soybean meal) and 56% starch (78.6% corn, 19% soybean meal, 2.4% urea) were selected. Starch intake was low with the 2% starch supplement representing approximately 0.15% of total organic matter intake whereas starch intake accounted for 8.6% of total organic matter intake when steers were fed the 56% starch supplement.

**Table 1:** Diet composition.

Item	Hay	2% Starch^1^	56% Starch^2^
**Chemical composition, % of DM**			
**Organic matter**	92.0	92.0	97.7
**Neutral detergent fiber**	74.4	10.0	14.5
**Acid detergent fiber**	44.0	6.6	4.4
**Crude protein**	3.5	43.0	21.2
**Protein degradability^3^, % of CP**		69.0	43.0

1 2% starch supplement= 100% soybean meal.

2 56% starch supplement= 78.6% cracked corn, 19% soybean meal, 2.4% urea.

3 Degradability determined via *Streptomyces griseus* method.

Experimental periods were 14 d long with 13 d for adaptation and ruminal samples were collected on d14. Experimental design and adaptation period length was chosen based on previous research in our lab and others (McCann et al. 2014, 2016; Latham et al. 2018; Clemmons et al. 2020). Ruminal fluid samples from the ventral sac were collected prior to feeding (h 0) and at 4 h post-feeding via the rumen cannula and strained through three layers of cheesecloth before being flash frozen in liquid nitrogen. Samples were stored at −20 °C until RNA isolation.

### Metatranscriptomic library preparation

Frozen ruminal fluid was thawed, and total RNA was extracted using an RNEasy Mini Kit (Qiagen, Hilden, Germany). Total RNA concentration was measured using a Qubit RNA Assay Kit with a Qubit 4.0 Fluorometer (Life Technologies, CA, USA). Frozen isolated RNA quality was evaluated using a PicoGreen fluorometer for concentration, a Nanodrop spectrophotometer for OD ratios, and a Fragment Analyzer for fragment size (Table S1). The samples were then rRNA depleted using the Illumina Ribo-Zero Plus rRNA Microbiome depletion kit. Remaining RNA was processed with a PerkinElmer NextFlex Rapid Directional RNA-Seq kit 2.0 to create the final sequencing libraries. Libraries underwent high-throughput, 2 × 150 bp paired-end sequencing on an Illumina NovaSeq 6000 S4 platform.

### Bioinformatics pipeline

**Figure 1:**
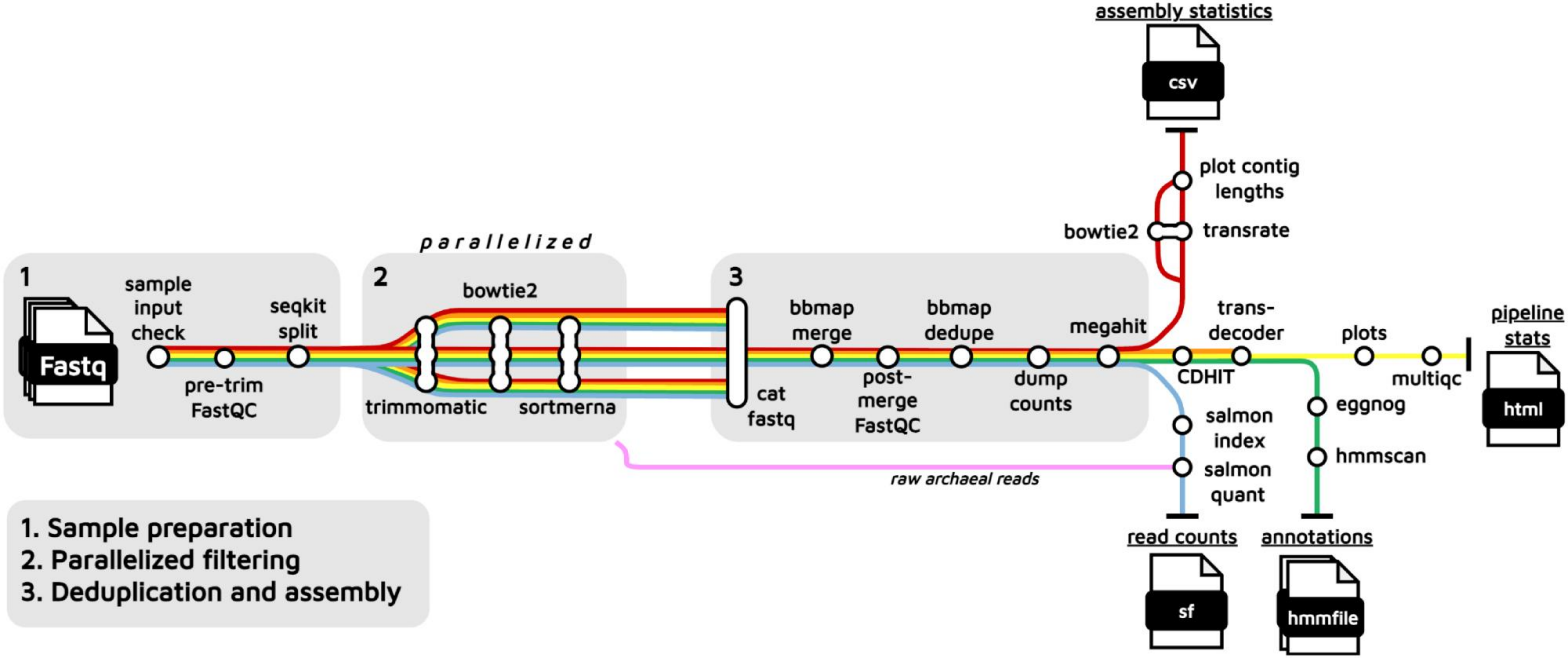
Bioinformatics pipeline used in the analysis.

A custom Nextflow pipeline (Di Tommaso et al. 2017) was used for bioinformatics analysis of the raw reads (Figure 1) and included all computational steps except for the differential expression analysis. First, a parallelized approach was used to split the large dataset into smaller units via seqkit v2.1.0 (Shen et al. 2024) for initial read processing and filtering. Raw reads were trimmed and filtered using Trimmomatic v0.39 and FastQC v0.12.1 (Simon et al. 2010; Bolger et al. 2014). MultiQC v1.14 (Ewels et al. 2016) was used to collate and summarize FastQC reports. Bowtie2 v2.4.4 and SortMeRNA v4.3.4 were used to remove bovine genomic reads and rRNA reads, respectively (Kopylova et al. 2012; Langmead and Salzberg 2012). Paired-end reads were merged using BBMerge (Bushnell et al. 2017) and deduplicated using BBmap v39.01 (Bushnell 2014). Sequences were assembled via Megahit v.1.2.9 (Li et al. 2015) and taxonomically classified via Kraken2 v2.1.2 (Wood et al. 2019). Reads were mapped back to the assembly using Bowtie2 v2.4.4 to assess assembly quality and the assembly run through Transrate v1.0.3 (Smith-Unna et al. 2016) to calculate assembly statistics. A quasi-mapping approach using salmon v1.10.1 (Patro et al. 2017) was also completed to estimate raw read abundance per transcript and produce a raw counts table.

Given the complexity of the metatranscriptome dataset, a large number of zeros were present in the raw counts table, either due to biological variability in gene expression between samples or technical limitations such as fragmented assembly contigs or insufficient sequencing depth. Therefore, a pseudocount of 1 was added to all raw read counts to prevent log transformation issues downstream. The counts matrix was then run through a separate pipeline (nf-core/differentialabundance v.1.2.0; Ewels et al. 2020; Wacker et al. 2023) for differential expression analysis. Briefly, the counts matrix was pre-filtered to only include genes with a minimum abundance of 2 reads in at least 1 sample. Differentially expressed genes were then identified using DESeq2 1.34.0 (Love et al. 2014) and the *P*value adjusted for false positives using the Benjamini-Hochberg correction method. Genes were considered significantly differentially expressed if they met the following criteria: a minimum |log2FoldChange| of 2, a p-adj < 0.05, and a baseMean of at least 10 to conservatively exclude genes with low read counts.

CD-HIT-EST v4.8.1 (Fu et al. 2012) was used to cluster any redundant assembly contigs and Transdecoder v5.5.0 (Haas and Papanicolaou 2018) was used to predict open reading frames and translate transcripts into peptide sequences. The assembly was functionally annotated using eggnog-mapper v2.1.9 and hmmscan v3.3.2 (Eddy 2011; Cantalapiedra et al. 2021) to predict function and taxa. Pathway enrichment analysis was conducted using the cluster Profiler R package (Yu et al. 2012), which mapped significant DEG to the Kyoto Encyclopedia of Genes and Genomes (KEGG; Kanehisa and Goto 2000) and used the DESeq2 1.34.0 results to determine enriched pathways and modules in each contrast.

Significant DEG (*P *< 0.05) were mapped to the carbohydrate active enzyme (CAZymes) database via eggnog-mapper v2.1.9 to determine expression of CAZymes in each contrast (Drula et al. 2022). To evaluate time and starch concentration effects, the number of significantly differentially expressed GH, GT, CBM, PL, and CE genes were counted for each sample. Statistical significance of CAZymes was analyzed using the PROC MIXED procedure in SAS 9.4 (SAS Institute Inc.), with time, starch concentration, and starch concentration × time as fixed effects and steer as a random effect.

## Results

Microbial RNA from ruminal fluid of steers fed LQF and two concentrations of starch supplementation (Table 1) was sequenced. Average sequencing depth was 200 million reads per sample (Table S1). From this, 2,379,275 contigs were assembled via Megahit (Li et al. 2015) with 28,123 contigs being classified as archaeal in origin via eggNOG-mapper. Reads were mapped back to the assembly using Bowtie2; 56.25% of reads mapped back to the assembly. A total of 2,727,644 peptides were annotated, with archaea accounting for 30,164 (1.1%) of annotated peptides.

### Archaeal gene expression

**Figure 2:**
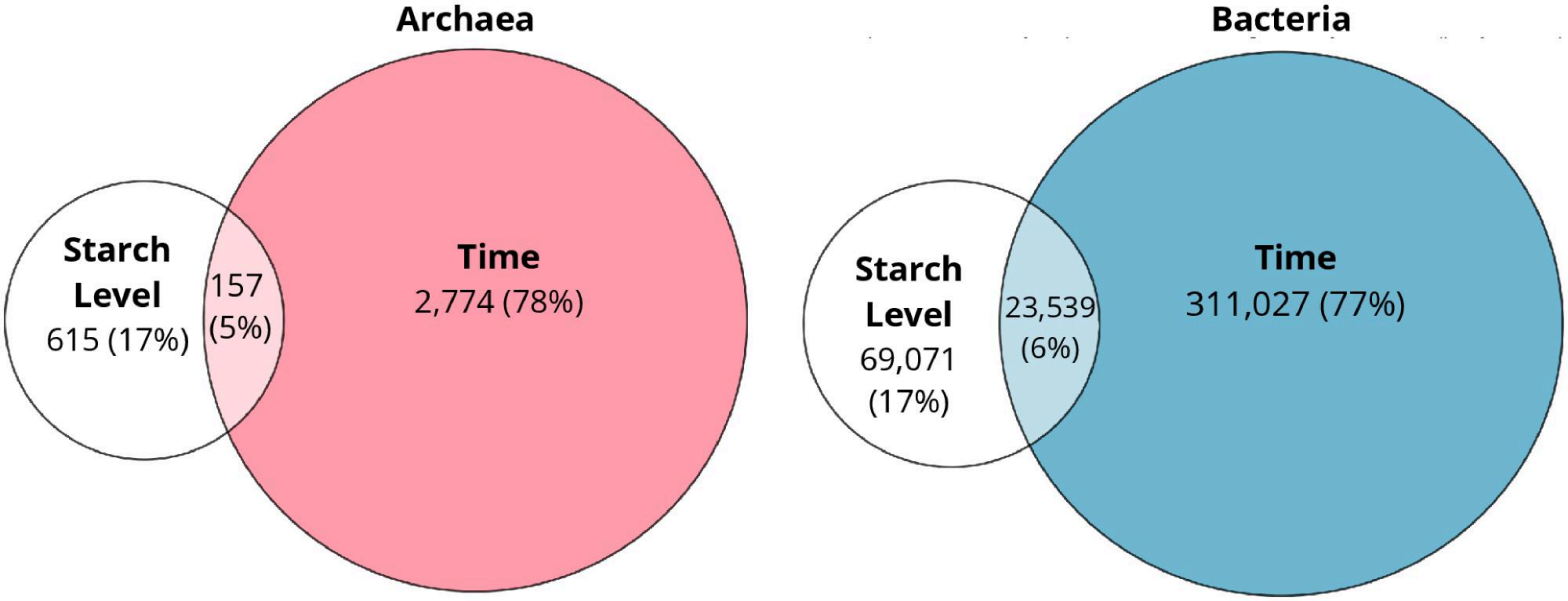
Overlap in differentially expressed genes by contrast (*P *< 0.05). Description. Proportion of differentially expressed genes that were affected by starch concentration (2 or 56%), time of sampling (h0 or 4 h post-supplementation), or both.

**Figure 3:**
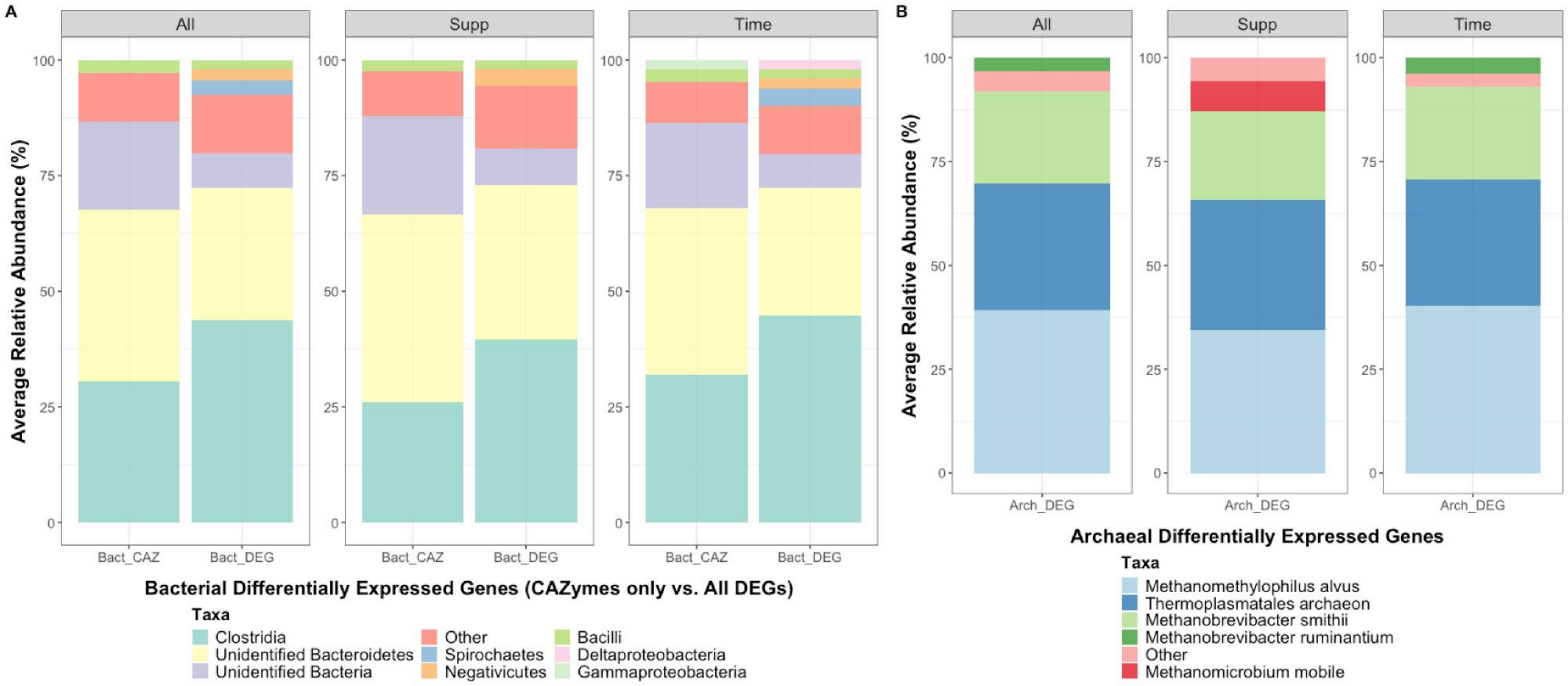
Average relative abundance of ruminal bacteria and archaea responsible for significant DEG. Description. Relative abundance of taxa responsible for all DEG, starch concentration (2 or 56% starch; *n* = 9), or time (h0 or 4 h post-supplementation; *n* = 9)

In archaea, 3,546 differentially expressed genes (DEG) were identified (*P *< 0.05) with time from feeding influencing expression of more genes than starch concentration (2,774 vs. 615 DEG, respectively; Figure 2). The majority of archaeal DEG involved in methanogenesis (95%) were expressed by two classes: Thermoplasmata (69.8%) and Methanobacteria (25.4%; Figure 3). Within the Thermoplasmata class, *Methanomethylophilus alvus* expressed 39.2% and members of Thermoplasmatales archaeon expressed 30.6% of archaeal DEG involved in methanogenesis. Within the Methanobacteria class, *Methanobrevibacter spp.* were responsible for all archaeal DEG involved in methanogenesis. Predominant enriched pathways were involved in global and overview maps (37%), amino acid metabolism (15%), and energy metabolism (13%; Table 2).

**Table 2:** Significantly enriched KEGG pathways of ruminal archaea.

Subcategory	Percentage
**Global and overview maps**	37%
** Biosynthesis of amino acids**	11%
** Biosynthesis of cofactors**	10%
** Carbon metabolism**	10%
** 2-oxocarboxylic acid metabolism**	4%
** Nucleotide metabolism**	2%
**Amino acid metabolism**	15%
**Energy metabolism**	13%
**Carbohydrate metabolism**	9%
**Translation**	7%
**Metabolism of cofactors and vitamins**	7%
**Nucleotide metabolism**	6%
**Replication and repair**	3%
**Folding, sorting, and degradation**	1%
**Metabolism of terpenoids and polyketides**	1%
**Other/unknown**	1%

**Figure 4:**
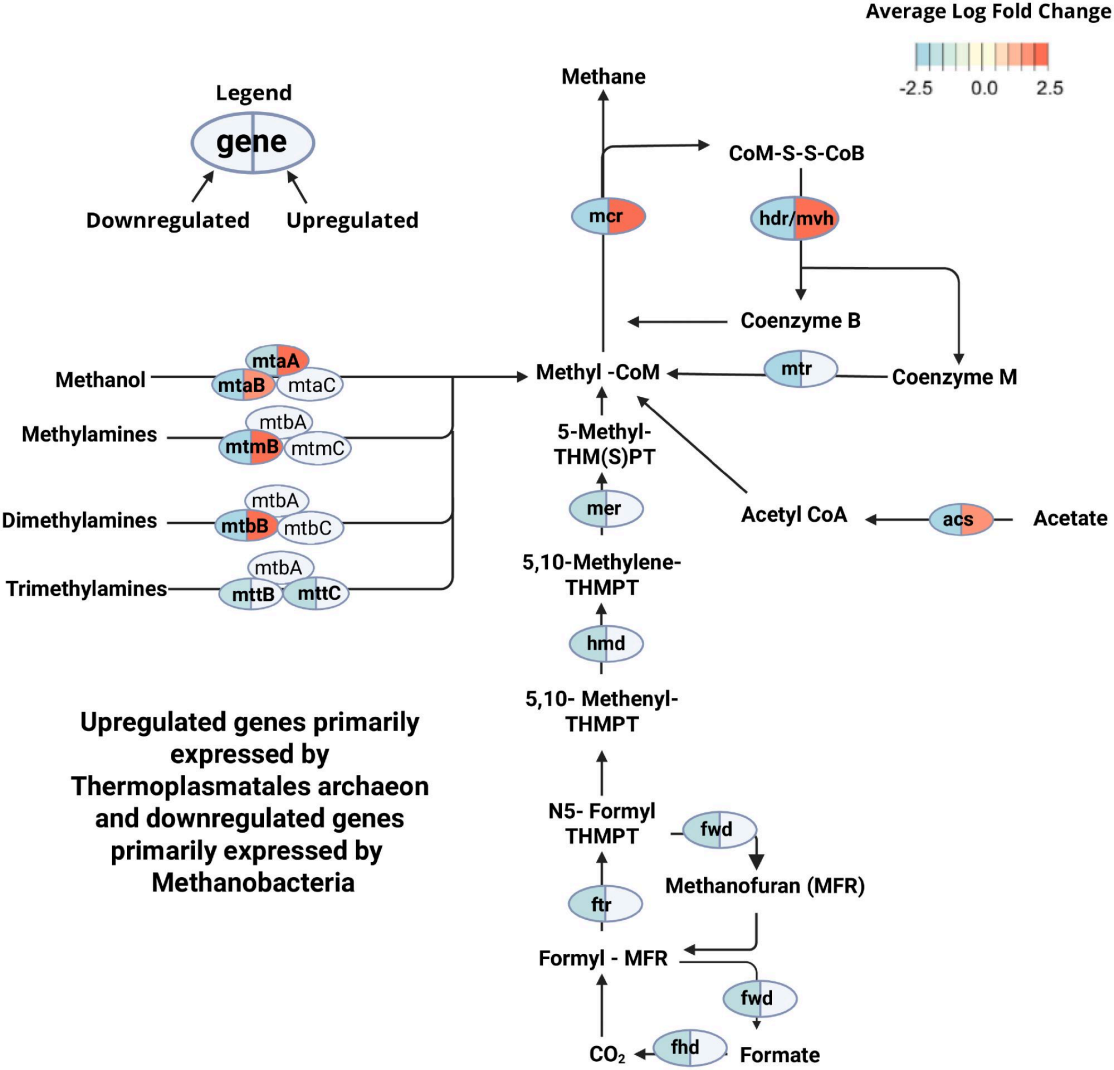
Differentially expressed archaeal genes involved in methanogenesis at 4 h post-feeding. Description: Methanogenic DEG affected by sampling hour (0 h or 4 h post-supplementation; *n* = 9). Enzymes in red indicate upregulation and enzymes in blue indicate downregulation at 4 h post-supplementation. Only archaeal DEG expressed by the two predominant classes, Thermoplasmata and Methanobacteria, are shown. Gene acronyms: acs: acetyl CoA synthetase, fdh: formate dehydrogenase, ftr: formylmethanofuran-tetrahydromethanopterin N-formyltransferase (multiple subunits), fwd: formylmethanofuran dehydrogenase, hdr: heterodisulfide reductase (multiple subunits), hmd: 5,10-methylenetetrahydromethanopterin hydrogenase, mcr: methyl-coenzyme M reductase (multiple subunits), mer: 5,10-methylenetetrahydromethanopterin reductase, mtaA/mtaB: methyltransferases, mtaC: methanol corrinoid protein, mtbA: methylamine corrinoid protein, mtbB: dimethylamine corrinoid protein co-methyltransferase, mtbC: dimethylamine corrinoid protein, mtmB: methaylamine-corrinoid protein comethyltransferase, mtmC: monomethylamine corrinoid protein, mtr: tetrahydromethanopterin S-methyltransferase (multiple subunits), mttB: trimethylamine-corrinoid protein co-methyltransferase, mttC: trimethylamine corrinoid protein, mvh: F420 non-reducing hydrogenase

Of the 2,774 archaeal DEG affected by time, 113 DEG were involved in methane metabolism with 45 DEG downregulated and 68 DEG upregulated at 4 h post-feeding (Table S2). All methanogenesis pathways were differentially regulated by time as a result of differences in gene expression by individual taxa (Figure 4). Genes common to all methanogenesis pathways, methyl coenzyme M reductase (*mcrA, mcrB*, and *mcrG*) and heterodisulfide reductase (*hdrA2* and *hdrD*) were both up and downregulated at 4 h post-feeding (*P *< 0.05). Additionally, genes exclusive to methylotrophic methanogenesis, methanol—5-hydroxybenzimidazolylcobamide Co-methyltransferase (*mtaB*), [methyl-Co(III) methanol/glycine betaine-specific corrinoid protein]: coenzyme M methyltransferase (*mtaA*), methylamine—corrinoid protein Co-methyltransferase (*mtmB*), and dimethylamine corrinoid protein Co-methyltransferase (*mtbB*), and one gene exclusive to acetoclastic methanogenesis, acetyl CoA synthetase (*acs*), were both up and downregulated at 4 h post-feeding (*P *< 0.05). As a result, hydrogenotrophic, methylotrophic, and acetoclastic methanogenesis pathways were both up and downregulated at 4 h post-feeding (*P *< 0.05). Upregulated genes were primarily expressed by members of the Thermoplasmata class, including a Thermoplasmatales archaeon and *Methanomethylophilus alvus*, a species of the order Methanomassiliicoccales. Downregulated genes were primarily expressed by members of the Methanobacteria class, *Methanobrevibacter spp.*

Additionally, *hdrB2* and *hdrC2*, which are common to all methanogenesis pathways, were upregulated at 4 h post-feeding (*P *< 0.05) and expressed by *Methanomethylophilus alvus*. Further, formate dehydrogenase (coenzyme F420) beta subunit (*fdhB*), a gene common to all methanogenesis pathways was downregulated at 4 h post-feeding (*P *< 0.05). Downregulated genes exclusive to hydrogenotrophic methanogenesis included: formylmethanofuran dehydrogenase subunits A and B (*fwdA* and *fwdB*), 5,10-methylenetetrahydromehanopterin reductase (*mer*), 5,10-methylenetetrahydromehanopterin hydrogenase (*hmd*), and formylmethanofuran-tetrahydromethanopterin N-formyltransferase (*ftr*). Downregulated genes common to all methanogenic pathways and the ones specific to the hydrogenotrophic pathway were primarily expressed by *Methanobrevibacter spp.* Additionally, downregulated genes exclusive to methylotrophic methanogenesis included: trimethylamine corrinoid protein Co-methyltransferase (*mttB*), trimethylamine corrinoid protein (*mttC*), and tetrahydromethanopterin S-methyltransferase subunits A, D, and H (*mtrA, mtrD*, and *mtrH*) and were expressed by members of Thermoplasmatales or Methanomicrobiales.

**Figure 5:**
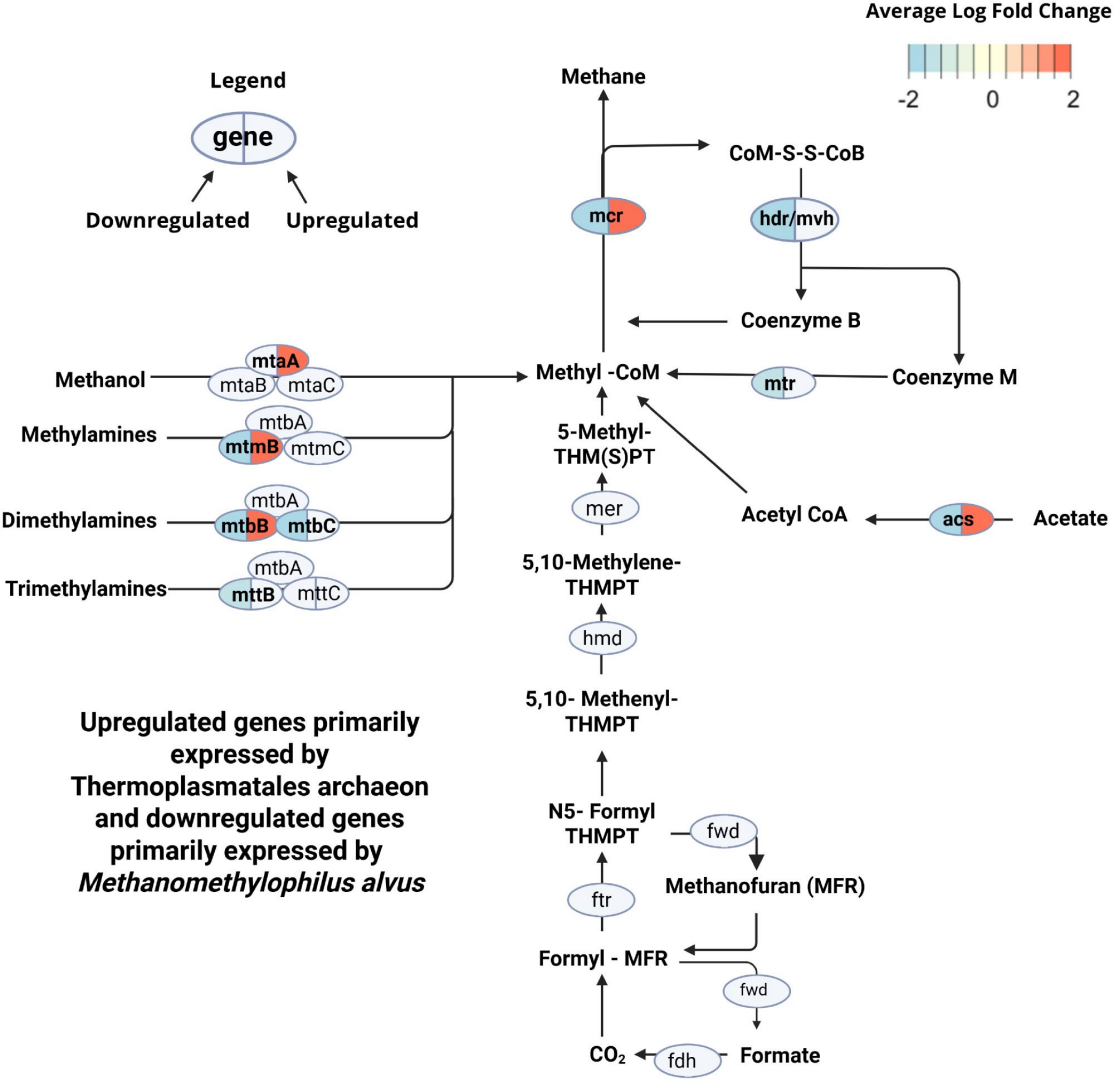
Differentially expressed archaeal genes involved in methanogenesis with the 56% starch supplement. Description: Methanogenic DEG affected by starch concentration (2 or 56% starch; *n* = 9). Enzymes in red indicate upregulation and enzymes in blue indicate downregulation in the 56% starch treatment. Only archaeal DEG expressed by the two predominant classes, Thermoplasmata and Methanobacteria, are shown. Gene acronyms: acs: acetyl CoA synthetase, fdh: formate dehydrogenase, ftr: formylmethanofuran-tetrahydromethanopterin N-formyltransferase (multiple subunits), fwd: formylmethanofuran dehydrogenase, hdr: heterodisulfide reductase (multiple subunits), hmd: 5,10-methylenetetrahydromethanopterin hydrogenase, mcr: methyl-coenzyme M reductase (multiple subunits), mer: 5,10-methylenetetrahydromethanopterin reductase, mtaA/mtaB: methyltransferases, mtaC: methanol corrinoid protein, mtbA: methylamine corrinoid protein, mtbB: dimethylamine corrinoid protein co-methyltransferase, mtbC: dimethylamine corrinoid protein, mtmB: methaylamine-corrinoid protein comethyltransferase, mtmC: monomethylamine corrinoid protein, mtr: tetrahydromethanopterin S-methyltransferase (multiple subunits), mttB: trimethylamine-corrinoid protein co-methyltransferase, mttC: trimethylamine corrinoid protein, mvh: F420 non-reducing hydrogenase

Of the archaeal DEG affected by starch concentration, 37 were involved in methane metabolism with 24 DEG downregulated in the 56% starch supplement and 13 DEG upregulated in the 56% starch supplement (Table S3). Genes coding for *mcrA, acs*, *mtmB, mttB*, and *mtbB* were both up and down regulated in the 56% starch supplement resulting in the up and down regulation of acetoclastic and methylotrophic methanogenesis (Figure 5; *P *< 0.05). Upregulated genes were primarily expressed by members of the Thermoplasmata class, Thermoplasmatales archaeon. Downregulated genes were primarily expressed by members of the Thermoplasmata class, *Methanomethylophilus alvus*.

The greater starch concentration resulted in the upregulation of *mcrB* (*P *< 0.05), which is common to all methanogenesis pathways. Other upregulated genes exclusive to methylotrophic methanogenesis included: *mtaA* and *mttC*, both being primarily expressed by Thermoplasmatales archaeon. Downregulated genes common to all methanogenesis pathways include: *mcrG, hdrA2, hdrC2*, and *mvhA*. Downregulated genes exclusive to hydrogenotrophic methanogenesis included: *fwdB, fwdF, fwdG*, and methylenetetrahydromethanopterin dehydrogenase (*mtd).* Dimethylamine corrinoid protein (*mtbC*), which is exclusive to methylotrophic methanogenesis, was downregulated. As a result, the hydrogenotrophic methanogenesis pathway was downregulated (*P *< 0.05) with the 56% starch supplement. The majority of downregulated DEG involved in hydrogenotrophic methanogenesis were expressed by *Methanomicrobium mobile.*

### Bacterial gene expression

In bacteria, 403,637 DEG (*P *< 0.05) were identified. Time resulted in more DEG than starch concentration (311,027 vs. 69,071 respectively). Clostridia expressed the majority of bacterial DEG in both contrasts (45 and 40% for time and starch concentration, respectively) followed by unclassified Bacteroidetes (28 and 33% for time and starch concentration, respectively). Predominant enriched pathways were involved in carbohydrate metabolism (16%), amino acid metabolism (13%), and metabolism of cofactors and vitamins (12%; Table 3).

**Table 3:** Significantly enriched KEGG pathways by ruminal bacteria.

Subcategory	Percentage
**Global and overview maps**	22%
** Biosynthesis of cofactors**	6%
** Biosynthesis of amino acids**	5%
** Carbon metabolism**	5%
** Biosynthesis of nucleotide sugars**	3%
** Other**	3%
**Carbohydrate metabolism**	20%
**Amino acid metabolism**	12%
**Metabolism of cofactors and vitamins**	8%
**Energy metabolism**	7%
**Glycan synthesis and metabolism**	4%
**Nucleotide metabolism**	4%
**Cellular community—prokaryotes**	4%
**Translation**	2%
**Replication and repair**	2%
**Metabolism of other amino acids**	2%
**Lipid metabolism**	2%
**Cell motility**	2%
**Membrane transport**	2%
**Drug resistance: antimicrobial**	2%
**Xenobiotics biodegradation and metabolism**	2%
**Folding, sorting, and degradation**	1%
**Other/unknown**	2%

A total of 13,144 DEG coded for one or more CAZymes, resulting in 14,108 differentially expressed CAZymes. Glycoside hydrolases were the most abundant CAZyme family observed, followed by GT, CBM, CE, and PL (Table 4). There were no supplementation × time interactions (*P *≥ 0.24) on the number of differentially expressed CAZymes. Total CAZymes and all CAZyme families were affected by time (*P *≤ 0.02) with increased number of gene counts at 4 h post-feeding compared to h0. The predominant taxa expressing CAZymes were unidentified bacteria belonging to the Bacteroidetes phylum (40 and 36% for supplement and time, respectively) followed by Clostridia (26 and 32% for supplement and time, respectively).

**Table 4:** Effect of starch supplementation and time on bacterial DEG that mapped as CAZymes.

	Treatment^1^					*P*-Value		
	2% Starch		56% Starch					
	0 h	4 h	0 h	4 h	SEM^2^	Starch	Time	Starch × Time
**Carbohydrate binding modules**	357	423	295	491	55.1	0.97	0.02	0.24
**Carbohydrate esterases**	208	207	163	232	34.4	0.75	0.28	0.29
**Glycoside hydrolases**	3,222	4,135	2,490	4,643	585.01	0.84	0.02	0.28
**Glycoside transferases**	1,432	1,889	1,130	2,083	238.8	0.81	< 0.01	0.29
**Polysaccharide lyases**	41	62	30	74	12.9	0.95	0.02	0.36
**Total**	5,261	6,717	4,107	7,522	919.9	0.84	0.02	0.28

1 2% starch = 100% soybean meal; 56% starch = 78.6% corn; 19% soybean meal; 2.4% urea.

2 Standard error of the mean.

## Discussion

Results of this experiment indicate that time relative to supplementation (prior to supplementation vs. 4 h post-supplementation) and supplemental starch concentration (2 vs. 56% starch) affected expression of archaeal genes involved in methanogenesis and bacterial genes involved in carbohydrate metabolism. Ruminal samples from the intermediate starch treatments (20 and 38% DM) were not analyzed. When interpreting these results, it is important to note that cattle were adapted to their respective supplements for 13 d prior to rumen sampling while receiving a consistent basal diet (King Ranch bluestem) throughout the study. This adaptation period has been used in similar studies (McCann et al. 2014, 2016; Latham et al. 2018; Clemmons et al. 2020); however, potential carryover effects from previous supplementation periods cannot be excluded. Despite this limitation, the predominance of significant time effects relative to supplementation, combined with the consistency of the basal diet, suggests these data provide meaningful insight into treatment responses. Additionally, 16S rRNA compositional data were not collected; therefore, the results reflect differential gene expression rather than shifts in ruminal prokaryotic composition.

### Archaeal gene expression

Time from supplementation, rather than starch concentration, elicited a greater effect on archaeal DEG. Postprandial variation in ruminal fermentation profiles in cattle consuming LQF and supplemental protein is well-documented. Supplementing protein to cattle consuming LQF increases FOMI, resulting in increased ruminal fill and available substrate for microbial fermentation (Mathis et al. 2000; Wickersham et al. 2004, 2008). As a result, bacterial fermentation and VFA concentrations increase (McCann et al. 2017). Van Lingen et al. (2017) observed peak archaea numbers 3 h post-feeding, suggesting increased methanogenic activity during this time as well.

While time of sampling relative to supplementation resulted in a difference in the number of DEG, it did not result in a difference in the number of enriched methanogenic pathways (*P *> 0.05). The majority of archaeal DEG affected by time are involved in all methanogenesis pathways, regardless of substrate (*mcrA, mcrB, mcrG, hdrA2, hdrB2, hdrC2, hdrD, mvhG*) resulting in the differential regulation of all three methanogenic pathways. Despite this, there was a clear taxa distinction between up and downregulated genes. Upregulated genes were expressed primarily by archaea belonging to the Thermoplasmata class, namely Thermoplasmatales archaeon and *Methanomethylophilus alvus* and downregulated genes expressed by *Methanobrevibacter spp*. Archaea belonging to the Thermoplasmata class are methylotrophic methanogens that primarily use methylamines as a carbon source (Poulsen et al. 2013; Zhou et al. 2021). In contrast, *Methanobrevibacter* are solely hydrogenotrophic methanogens, using CO_2_ or formate as a carbon source. Increased expression by methylotrophic methanogens 4 h post-supplementation may be driven by the dietary conversion of choline to trimethylamines, providing abundant substrate for methylotrophic methanogenesis (Zhou et al. 2021). Rapid conversion of choline to trimethylamines by anaerobic bacteria is well-documented in monogastrics and ruminants (Neill et al. 1978; Craciun and Balskus 2012; Wang et al. 2012). Additionally, labeled methyl groups of choline have been observed to be metabolized into CH_4_ with trimethylamine as an intermediate in the rumen (Neill et al. 1978). In the present study, steers consumed an average of 1,413 mg of choline at 0 h in the form of soybean meal and corn (Menten et al. 1997; INRAE). Therefore, the increased activity of methylotrophic archaea 4 h post-feeding is potentially the result of increased substrate availability 4 h post-feeding. Similar to our observation, Söllinger et al. (2018) observed increased mRNA transcripts of methylotrophic methanogens, but not hydrogenotrophic methanogens, 3 h post-feeding in dairy cattle fed a total mixed ration. Accordingly, methylotrophs potentially outcompete hydrogenotrophs in the first 4 h post-feeding. However, in the present study, archaeal community composition was not measured. More research, combining both compositional and gene expression data, is needed to better understand the dynamics of the competition between various classes of ruminal methanogenic archaea in response to feeding.

Methylotrophic and acetoclastic methanogenesis pathways were both up and downregulated by the 56% starch supplement as result of differential expression by taxa. Both up and downregulated genes were primarily expressed by archaea belonging to the Thermoplasmata class. However, Thermoplasmatales archaeon was primarily responsible for the upregulated genes and *Methanomethylophilus alvus*, a member of the order Methanomassiliicoccales, was primarily responsible for the expression of downregulated genes. It is unclear as to why increased starch concentration resulted in differential expression of Thermoplasmata members. However, Teobaldo et al. (2023) observed an increase in the relative abundance of Thermoplasmatales in grazing beef cattle when they were supplemented with an energy supplement (0.3% BW corn gluten meal) compared to no energy supplement. In the present study, steers on the 56% starch supplement consumed an average of 0.3% BW of corn, soybean meal, and urea. Additionally, Jin et al. (2017) utilized real-time PCR to observe decreased abundance of Methanomassiliicoccales in a grain-based diet compared to a forage-based diet (0.5 vs. 8% of ruminal archaea abundance). Therefore, Thermoplasmatales may outcompete *Methanomethylophilus alvus*, subsequently playing a larger role when starch is provided to cattle consuming forage-based diets. It is worth noting that *Methanomethylophilus alvus* has been successfully isolated from human feces but not the rumen. Additionally, differences in gene expression may not correlate to differences in community composition. Further research incorporating both DNA and RNA approaches is necessary to improve our understanding of the relationship between *M. alvus* and Thermoplasmatales in the rumen.

The acetoclastic methanogenesis pathway was also differentially regulated, primarily due to genes expressed by *Methanomethylophilus alvus* and Thermoplasmatales. The slow turnover rate of acetoclastic methanogens renders acetoclastic methanogenesis a negligible pathway in the rumen (Wolfe 1982; Janssen and Kirs 2008). The majority of archaeal DEG affected by supplementation involved in acetoclastic methanogenesis are involved in the shared final methanogenesis reactions (*mcrA, mcrB, mcrG, hdrA2, hdrC2*, and *mvhA*) rather than acetoclastic methanogenesis exclusively. Further, the only archaeal DEG involved in methanogenesis that is specific to the acetoclastic pathway was acetyl CoA synthetase (*acs*) which was expressed by archaea belonging to either Thermoplasmata or Methanomicrobia. Members of Thermoplasmata have only been observed to use methylotrophic methanogenesis (Poulsen et al. 2013); however, two novel orders belonging to the Thermoplasmata class, *Candidatus* Sysuiplasmatales and Gimiplasmatales, have been observed to utilize *acs* for acetogenesis via the Wood-Ljungdahl pathway (Hu et al. 2021; Yuan et al. 2021). Additionally, certain members of Methanomicrobia, namely *Methanosarcina*, are capable of acetoclastic methanogenesis. While *Methanosarcina* was not specifically identified as expressing *acs* in the time contrast, there is the potential that another closely related methanogen belonging to the Methanomicrobia class expressed *acs* as part of acetoclastic methanogenesis. Further, as this discussion relies on sequence-based taxonomic and functional annotations which can be limited, more research investigating acetoclastic members of Methanomicrobia and the acetogenic members of Thermoplasmata in the rumen is warranted.

It is unclear why hydrogenotrophic methanogenesis was downregulated with the 56% starch supplement as it is the predominant methanogenesis pathway in the rumen (Greening et al. 2019). The downregulated archaeal DEG that are unique to the hydrogenotrophic pathway were *fwdA, fwdB, fwdF*, and *fwdG*, all of which are involved in the reduction of CO_2_ into formyl-methanofuran, a critical step in the hydrogenotrophic pathway. *Methanomicrobium mobile* was the primary taxa expressing these downregulated genes. Therefore, *Methanomicrobium mobile* may be more sensitive to increasing starch supplementation compared to other methanogen species. Both *in vivo* and *in vitro* studies have observed that high-grain diets (65% grain) affect the redox potential of *Methanomicrobium mobile*, resulting in decreased abundance in the high-grain compared to low-grain (0% grain) diets in dairy cattle (Friedman et al. 2017). More research investigating the response of *Methanomicrobium mobile* to starch supplementation is warranted.

### Bacterial gene expression

Similar to archaeal DEG, time exerted a greater effect compared to starch concentration on bacterial CAZyme expression. Postprandial variation in ruminal fermentation is well-documented with peak activity observed 3–4 h post-feeding in both high and low forage diets (Lewis 1957; Evans et al. 1975; Martin et al. 1993). Protein supplementation to cattle consuming LQF is well-documented to increase ruminal ammonia concentrations, increasing bacterial growth and fermentation (Wickersham et al. 2004, 2008). Therefore, it is not surprising that the number of differentially expressed CAZymes increased 4 h post-feeding. Diet has been observed to affect expression of bacterial CAZymes. Wang et al. (2019) collected ruminal fluid from cattle consuming either a high-forage or low-forage diet at 0 and 4 h post-feeding and observed greater expression of GT in the low forage and greater expression of CE in the high forage. However, in the present study, all diets were LQF-based with the supplements contributing 7–15% of the total organic matter intake. The provision of isonitrogenous supplements may have prevented significant differences in CAZyme expression between supplements.

Bacterial DEG were primarily expressed by Clostridia, followed by unclassified Bacteroidetes. Comtet-Marre et al. (2017) and Li and Guan (2017) observed similar results with Firmicutes being the predominant phylum identified using RNA sequencing in cattle consuming 50:50 concentrate: forage total mixed ration and a finishing ration, respectively. Bacteroidetes, specifically the genus *Prevotella*, is commonly observed as the predominant taxa in the rumen (Poulsen et al. 2013; Li et al. 2020; O’Hara et al. 2020). Therefore, the decreased differential gene expression of Bacteroidetes was unexpected. One theory for this observation is KR Bluestem is dependent on the C_4_ pathway, and C_4_ grasses are associated with greater amounts of cell-wall, specifically lignin, reducing the efficacy of mastication and inhibiting microbial cell-wall degradation compared to C_3_ grasses (Wilson 1994). Utilization of the nutrients entangled in the lignin-rich cell wall may require specialized lignocellulose degraders capable of producing an extracellular, multienzyme complex known as the cellulosome. Briefly explained, the microbial cell surface has anchoring protein domains composed of surface layer homology (SLH) bound to Type II cohesin domains (Jiang et al. 2021). Scaffolding proteins possess Type I cohesin and dockerin domains that bind cellulases to the scaffold (Jiang et al. 2021). The binding of Type II cohesin and Type II dockerin bind the anchoring protein and scaffolding proteins forming a complete cellulosome. Only certain members of the Firmicutes phylum, namely the class Clostridia, are capable of producing the cellulosome (Gharechahi et al. 2021). In the present study, 2,369 bacterial DEG coded for the SLH, cohesin, or dockerin proteins and 86% of those DEG were expressed by Clostridia. Further research is needed to better describe fiber degradation, cellulase activity, and microbial composition in the rumen.

Glycoside hydrolases were the most abundant CAZyme family observed in both the time and starch concentration contrast. Neves et al. (2021) also observed predominant GH abundance (42.2% of CAZyme matches) in cattle consuming a corn silage-based diet. In the present study, unclassified Bacteroidetes were responsible for the majority of CAZyme expression. Wang et al. (2019) observed *Prevotella*, belonging to the phylum Bacteroidetes, as the primary bacteria involved in CAZyme expression in cattle consuming both high-forage and low-forage diets. Additionally, *Prevotella* is considered the most abundant genus in ruminal contents, across diet, environment, or host species (O’Hara et al. 2020) supporting the theory of Bacteroidetes being the primary bacteria involved in differential gene expression of CAZymes.

## Conclusion

Time of sample collection after feeding, rather than starch concentration, elicited a greater effect on bacterial and archaeal gene expression in steers consuming LQF and supplemented protein. All methanogenic pathways were differentially regulated by time and taxa. Genes downregulated at h4 were expressed primarily by *Methanobrevibacter*, and genes upregulated at h4 were expressed primarily by Thermoplasmatales. Methylotrophic and acetoclastic methanogenesis were differentially expressed in response to starch concentration and taxa. Genes downregulated by the 56% starch supplement were primarily expressed by *Methanomethylophilus alvus* and upregulated genes were primarily expressed by Thermoplasmatales archaeon. Bacterial genes were primarily expressed by Clostridia, potentially due to the lignin-rich forage in the diet. Further research is needed to determine if differential expression of archaeal methanogenesis genes is associated with microbial composition and CH_4_ emissions. These findings demonstrate improvements in LQF utilization, with protein and starch supplementation, are correlated to relevant changes in gene expression of ruminal microbiota. Furthermore, they demonstrate that supplementation affects expression of genes involved in methanogenesis reactions, suggesting an opportunity to affect CH_4_ production. Studies quantifying forage utilization, ruminal microbiota gene expression, and CH_4_ production are needed to optimize supplementation strategies for improved beef production efficiency whilst maintaining or reducing its carbon footprint.

## Supplementary Material

skag188_Supplementary_Data

## Data Availability

Sequences have been submitted to the Sequence Read Archive (SRA) of the National Center for Biotechnology (NCBI) under the BioProject accession number PRJNA1338562. All other pertinent data are included in the manuscript and supplemental tables.

## Abbreviations

Acs: acetyl coA synthetase
AUX: auxiliary actions
BW: body weight
CAZyme: carbohydrate active enzyme
CBM: carbohydrate binding module
CE: carbohydrate esterase
CH_4_: methane
CO_2_: carbon dioxide
CoB: coenzyme B
CoM: coenzyme M
CP: crude protein
DEG: differentially expressed gene
DM: dry matter
*Fdh*: formate dehydrogenase
FOMI: forage organic matter intake
*Ftr*: formylmethanofuran-tetrahydromethanopterin N-formyltransferase
*Fwd*: formylmethanofuran dehydrogenase
GH: glycoside hydrolase
GT: glycoside transferase
*Hdr*: heterodisulfide reductase
*Hmd*: 5,10-methylenetetrahydromehanopterin hydrogenase
*KEGG*: Kyoto Encyclopedia of Genes and Genomes
LQF: low-quality forage
MCP: microbial crude protein
*Mcr*: methyl-coenzyme M reductase
*Mer*: 5,10-methylenetetrahydromehanopterin reductase
*MtaA*: [methyl-Co(III) methanol/glycine betaine-specific corrinoid protein]: Coenzyme M methyltransferase
*MtaB*: methanol—5-hydroxybenzimidazolylcobamide Co-methyltransferase
*MtbB*: dimethylamine corrinoid protein co-methyltransferase
*MtbC*: dimethylamine corrinoid protein
*Mtd*: methylenetetrahydromethanopterin dehydrogenase
*MtmB*: methylamine corrinoid protein Co-methyltransferase
*Mtr*: tetrahydromethanopterin S-methyltransferase
*MttB*: trimethylamine corrinoid protein Co-methyltransferase
*MttC*: trimethylamine corrinoid protein
*Mvh*: F420 non-reducing hydrogenase
PL: polysaccharide lyase
VFA: volatile fatty acid
