# Supplementary material for: Ruminal archaea and bacteria metatranscriptomic responses to supplementation in steers fed low-quality forage
Source: J Anim Sci. 2026 Jun 12;104:skag188. doi: 10.1093/jas/skag188 (PMC13317134; doi:10.1093/jas/skag188)
Supplement: skag188_Supplementary_Data [file skag188_supplementary_data.zip › Supplemental_Table_3.pdf]

Supplemental Table 3. Differentially expressed archaeal genes involved in methanogenesis with the 56% starch supplement

Gene Symbol	KO Term	Taxonomy	Log Fold Change	*P*-value
Upregulated with the 56% starch supplement				
Involved in KEGG Module M00356				
mtaA	K14080	*Methanomethylophilus alvus*	2.55	< 0.01
mcrB	K00401	*Methanomethylophilus alvus*	2.02	0.01
mcrA	K00399	*Methanobrevibacter smithii*	1.96	0.01
mcrB	K00401	Thermoplasmatales archaeon	1.64	0.02
mtaA	K14080	Thermoplasmatales archaeon	1.59	0.04
mcrB	K00401	Thermoplasmatales archaeon	1.56	0.04
Involved in KEGG Module M00357				
mcrB	K00401	*Methanomethylophilus alvus*	2.02	0.01
mcrA	K00399	*Methanobrevibacter smithii*	1.96	0.01
acs	K01895	*Methanomethylophilus alvus*	1.77	0.02
acs	K01895	*Methanocorpusculum labreanu*	1.76	0.02
mcrB	K00401	Thermoplasmatales archaeon	1.64	0.02
acs	K01895	Thermoplasmatales archaeon	1.58	0.04
mcrB	K00401	Thermoplasmatales archaeon	1.56	0.04
Involved in KEGG Module M00563				
mttC	K14084	*Methanosarcina mazei*	2.06	0.01
mcrB	K00401	*Methanomethylophilus alvus*	2.02	0.01
mcrA	K00399	*Methanobrevibacter smithii*	1.96	0.01
mttB	K14083	*Methanolobus psychrophilus*	1.88	0.01
mtmB	K16176	Thermoplasmatales archaeon	1.73	0.02
mtbB	K16178	*Methanomethylophilus alvus*	1.70	0.03
mcrB	K00401	Thermoplasmatales archaeon	1.64	0.02
mcrB	K00401	Thermoplasmatales archaeon	1.56	0.04
Involved in KEGG Module M00567				
mcrB	K00401	*Methanomethylophilus alvus*	2.02	0.01
mcrA	K00399	*Methanobrevibacter smithii*	1.96	0.01
mcrB	K00401	Thermoplasmatales archaeon	1.64	0.02
mcrB	K00401	Thermoplasmatales archaeon	1.56	0.04
Downregulated with the 56% starch supplement				
Involved in KEGG Module M00356				
hdrC2	K03390	*Methanomethylophilus alvus*	-1.91	0.01
mcrA	K00399	*Methanobrevibacter smithii*	-1.83	0.02
mcrG	K00402	*Methanomethylophilus alvus*	-1.76	0.02
hdrA2	K03388	Thermoplasmatales archaeon	-1.59	0.04
mcrA	K00399	*Methanobrevibacter smithii*	-1.56	0.04
mvhA	K14126	*Methanomethylophilus alvus*	-1.54	0.05
Involved in KEGG Module M00357				
acs	K01895	*Methanospirillum hungatei*	-1.93	0.01
hdrC2	K03390	*Methanomethylophilus alvus*	-1.91	0.01

acs	K01895	Thermoplasmatales archaeon	-1.88	0.01
mcrA	K00399	*Methanobrevibacter smithii*	-1.83	0.02
mcrG	K00402	*Methanomethylophilus alvus*	-1.76	0.02
mtrA	K00577	*Methanobrevibacter smithii*	-1.61	0.04
hdrA2	K03388	Thermoplasmatales archaeon	-1.59	0.04
mcrA	K00399	*Methanobrevibacter smithii*	-1.56	0.04
mvhA	K14126	*Methanomethylophilus alvus*	-1.54	0.05
Involved in KEGG Module M00563				
mtbB	K16178	*Methanomethylophilus alvus*	-2.24	< 0.01
mtbC	K16179	*Methanomethylophilus alvus*	-2.08	0.01
mtbB	K16178	*Methanomethylophilus alvus*	-1.98	0.01
mtmB	K16176	Thermoplasmatales archaeon	-1.92	0.01
hdrC2	K03390	*Methanomethylophilus alvus*	-1.91	0.01
mtbB	K16178	*Methanomethylophilus alvus*	-1.85	0.01
mtbC	K16179	*Methanomethylophilus alvus*	-1.85	0.01
mcrA	K00399	*Methanobrevibacter smithii*	-1.83	0.02
mcrG	K00402	*Methanomethylophilus alvus*	-1.76	0.02
mttB	K14083	*Methanomethylophilus alvus*	-1.59	0.04
hdrA2	K03388	Thermoplasmatales archaeon	-1.59	0.04
mcrA	K00399	*Methanobrevibacter smithii*	-1.56	0.04
mvhA	K14126	*Methanomethylophilus alvus*	-1.54	0.05
mtmB	K16176	Thermoplasmatales archaeon	-1.53	0.05
Involved in KEGG Module M00567				
fwdB	K00201	*Methanomicrobium mobile*	-2.21	< 0.01
fwdF	K00205	*Methanomicrobium mobile*	-1.93	0.01
fwdG	K11260	*Methanomicrobium mobile*	-1.93	0.01
hdrC2	K03390	*Methanomethylophilus alvus*	-1.91	0.01
mcrA	K00399	*Methanobrevibacter smithii*	-1.83	0.02
mcrG	K00402	*Methanomethylophilus alvus*	-1.76	0.02
fwdA	K00200	*Methanomicrobium mobile*	-1.75	0.02
mtd	K00319	*Methanomicrobium mobile*	-1.74	0.02
fwdA	K00200	*Methanobrevibacter smithii*	-1.70	0.03
mtrA	K00577	*Methanobrevibacter smithii*	-1.61	0.04
hdrA2	K03388	Thermoplasmatales archaeon	-1.59	0.04
mcrA	K00399	*Methanobrevibacter smithii*	-1.56	0.04
mvhA	K14126	*Methanomethylophilus alvus*	-1.54	0.05
fwdB	K00201	*Methanomicrobium mobile*	-1.49	0.05
