# Supplementary material for: Ruminal archaea and bacteria metatranscriptomic responses to supplementation in steers fed low-quality forage
Source: J Anim Sci. 2026 Jun 12;104:skag188. doi: 10.1093/jas/skag188 (PMC13317134; doi:10.1093/jas/skag188)
Supplement: skag188_Supplementary_Data [file skag188_supplementary_data.zip › Supplemental_Table_2.pdf]

Supplemental Table 2. Differentially expressed archaeal genes involved in methanogenesis at 4h post-feeding

Gene Symbol	KO Term	Taxonomy	Log Fold Change	P-value
Archaeal DEGS upregulated at H4				
Involved in KEGG Module M00356				
mcrB	K00401	Thermoplasmatales archaeon	4.28647	< 0.01
mtaA	K14080	*Methanomethylophilus alvus*	3.43113	< 0.01
mcrA	K00399	Thermoplasmatales archaeon	3.080039	< 0.01
mcrB	K00401	Thermoplasmatales archaeon	3.015968	< 0.01
mcrA	K00399	*Methanomethylophilus alvus*	2.867172	< 0.01
hdrC2	K03390	*Methanomethylophilus alvus*	2.839452	< 0.01
mtaA	K14080	*Methanomethylophilus alvus*	2.816199	< 0.01
mtaB	K04480	*Methanomethylophilus alvus*	2.764931	< 0.01
mcrB	K00401	Thermoplasmatales archaeon	2.751106	< 0.01
mtaA	K14080	Thermoplasmatales archaeon	2.512799	< 0.01
hdrD	K08264	*Methanomethylophilus alvus*	2.416595	< 0.01
mcrA	K00399	*Methanomethylophilus alvus*	2.38888	0.01
mcrA	K00399	Thermoplasmatales archaeon	2.233378	0.01
mcrA	K00399	*Methanomethylophilus alvus*	2.227565	0.00
mcrA	K00399	*Methanomethylophilus alvus*	2.201209	0.01
mcrA	K00399	*Methanomethylophilus alvus*	2.180225	0.01
mtaA	K14080	Thermoplasmatales archaeon	2.166089	0.01
mtaA	K14080	*Methanomethylophilus alvus*	2.119553	0.01
mcrA	K00399	*Methanomethylophilus alvus*	2.116668	0.01
mcrB	K00401	Thermoplasmatales archaeon	2.08649	0.01
mcrA	K00399	Thermoplasmatales archaeon	2.060044	0.01
mcrA	K00399	*Methanomethylophilus alvus*	2.026371	0.01
mcrB	K00401	Thermoplasmatales archaeon	1.97292	0.02
mcrA	K00399	*Methanomethylophilus alvus*	1.970599	0.02
mcrA	K00399	Thermoplasmatales archaeon	1.966109	0.02
mvhG	K14128	*Methanomethylophilus alvus*	1.95798	0.02
mcrA	K00399	*Methanomethylophilus alvus*	1.95451	0.01
mtaA	K14080	Thermoplasmatales archaeon	1.929281	0.03
mtaA	K14080	*Methanomethylophilus alvus*	1.923878	0.02
mcrA	K00399	Thermoplasmatales archaeon	1.901289	0.01
hdrA2	K03388	Thermoplasmatales archaeon	1.880038	0.02
mcr	K00399	Thermoplasmatales archaeon	1.86647	0.03
mcrB	K00401	*Methanomethylophilus alvus*	1.864139	0.03
mcrG	K00402	Thermoplasmatales archaeon	1.8376	0.02
hdrA2	K03388	Thermoplasmatales archaeon	1.829351	0.04
mcrB	K00401	Thermoplasmatales archaeon	1.810528	0.03
mcrA	K00399	*Methanomethylophilus alvus*	1.807524	0.02
mcrG	K00402	*Methanomethylophilus alvus*	1.801436	0.04
mcrB	K00401	Thermoplasmatales archaeon	1.770492	0.02
mcrA	K00399	*Methanomethylophilus alvus*	1.760893	0.02

hdrB2	K03389	*Methanomethylophilus alvus*	1.756497	0.04
mcrA	K00399	*Methanomethylophilus alvus*	1.752823	0.03
mcrB	K00401	Thermoplasmatales archaeon	1.746377	0.04
hdrD	K08264	*Methanomethylophilus alvus*	1.728693	0.03
mcrG	K00402	*Methanomethylophilus alvus*	1.727742	0.05
mcrA	K00399	Thermoplasmatales archaeon	1.726943	0.04
mtaA	K14080	*Methanomethylophilus alvus*	1.714151	0.05
mcrA	K00399	*Methanomethylophilus alvus*	1.712247	0.04
mcrA	K00399	*Methanomethylophilus alvus*	1.67934	0.05
mcrA	K00399	*Methanomethylophilus alvus*	1.667273	0.04
hdrD	K08264	*Methanomethylophilus alvus*	1.656392	0.05
mtaB	K04480	*Methanomethylophilus alvus*	1.57	0.05
mcrA	K00399	*Methanomethylophilus alvus*	1.407736	0.05
Involved in KEGG Module M00357				
mcrB	K00401	Thermoplasmatales archaeon	4.28647	< 0.01
acs	K01895	Thermoplasmatales archaeon	3.244942	< 0.01
mcrA	K00399	Thermoplasmatales archaeon	3.080039	< 0.01
mcrB	K00401	Thermoplasmatales archaeon	3.015968	< 0.01
mcrA	K00399	*Methanomethylophilus alvus*	2.867172	< 0.01
hdrC2	K03390	*Methanomethylophilus alvus*	2.839452	< 0.01
mcrB	K00401	Thermoplasmatales archaeon	2.751106	< 0.01
acs	K01895	*Methanolacinia petrolearia*	2.44708	< 0.01
hdrD	K08264	*Methanomethylophilus alvus*	2.416595	< 0.01
mcrA	K00399	*Methanomethylophilus alvus*	2.38888	< 0.01
mcrA	K00399	Thermoplasmatales archaeon	2.233378	< 0.01
mcrA	K00399	*Methanomethylophilus alvus*	2.227565	0.01
mcrA	K00399	*Methanomethylophilus alvus*	2.201209	0.01
mcrA	K00399	*Methanomethylophilus alvus*	2.180225	0.01
acs	K01895	Thermoplasmatales archaeon	2.141498	0.01
mcrA	K00399	*Methanomethylophilus alvus*	2.116668	0.01
mcrB	K00401	Thermoplasmatales archaeon	2.08649	0.01
mcrA	K00399	Thermoplasmatales archaeon	2.060044	0.01
mcrA	K00399	*Methanomethylophilus alvus*	2.026371	0.01
acs	K01895	*Methanomicrobium mobile*	1.989739	0.02
acs	K01895	Thermoplasmatales archaeon	1.980357	0.02
mcrB	K00401	Thermoplasmatales archaeon	1.97292	0.02
mcrA	K00399	*Methanomethylophilus alvus*	1.970599	0.02
mcrA	K00399	Thermoplasmatales archaeon	1.966109	0.02
mvhG	K14128	*Methanomethylophilus alvus*	1.95798	0.02
mcrA	K00399	*Methanomethylophilus alvus*	1.95451	0.01
mcrA	K00399	Thermoplasmatales archaeon	1.901289	0.01
hdrA2	K03388	Thermoplasmatales archaeon	1.880038	0.02
acs	K01895	Thermoplasmatales archaeon	1.8735	0.02
acs	K01895	*Methanocorpusculum labreanum*	1.86881	0.03

mcrA	K00399	Thermoplasmatales archaeon	1.86647	0.03
mcrB	K00401	*Methanomethylophilus alvus*	1.864139	0.03
mcrG	K00402	Thermoplasmatales archaeon	1.8376	0.02
hdrA2	K03388	Thermoplasmatales archaeon	1.829351	0.04
mcrB	K00401	Thermoplasmatales archaeon	1.810528	0.03
mcrA	K00399	*Methanomethylophilus alvus*	1.807524	0.02
mcrG	K00402	*Methanomethylophilus alvus*	1.801436	0.04
mcrB	K00401	Thermoplasmatales archaeon	1.770492	0.02
mcrA	K00399	*Methanomethylophilus alvus*	1.760893	0.02
hdrB2	K03389	*Methanomethylophilus alvus*	1.756497	0.04
mcrA	K00399	*Methanomethylophilus alvus*	1.752823	0.03
mcrB	K00401	Thermoplasmatales archaeon	1.746377	0.04
hdrD	K08264	*Methanomethylophilus alvus*	1.728693	0.03
mcrG	K00402	*Methanomethylophilus alvus*	1.727742	0.05
mcrA	K00399	Thermoplasmatales archaeon	1.726943	0.04
mcrA	K00399	*Methanomethylophilus alvus*	1.712247	0.04
mcrA	K00399	*Methanomethylophilus alvus*	1.67934	0.05
mcrA	K00399	*Methanomethylophilus alvus*	1.667273	0.04
hdrD	K08264	*Methanomethylophilus alvus*	1.656392	0.05
mcrA	K00399	*Methanomethylophilus alvus*	1.407736	0.05
Involved in KEGG Module M00563				
mcrB	K00401	Thermoplasmatales archaeon	4.28647	< 0.01
mcrA	K00399	Thermoplasmatales archaeon	3.080039	< 0.01
mcrB	K00401	Thermoplasmatales archaeon	3.015968	< 0.01
mcrA	K00399	*Methanomethylophilus alvus*	2.867172	< 0.01
hdrC2	K03390	*Methanomethylophilus alvus*	2.839452	< 0.01
mcrB	K00401	Thermoplasmatales archaeon	2.751106	< 0.01
mtmB	K16176	Thermoplasmatales archaeon	2.488639	0.01
hdrD	K08264	*Methanomethylophilus alvus*	2.416595	0.01
mcrA	K00399	*Methanomethylophilus alvus*	2.38888	0.01
mtmB	K16176	*Methanomethylophilus alvus*	2.349845	0.01
mcrA	K00399	Thermoplasmatales archaeon	2.233378	0.01
mcrA	K00399	*Methanomethylophilus alvus*	2.227565	0.01
mcrA	K00399	*Methanomethylophilus alvus*	2.201209	0.01
mcrA	K00399	*Methanomethylophilus alvus*	2.180225	0.01
mcrA	K00399	*Methanomethylophilus alvus*	2.116668	0.01
mcrB	K00401	Thermoplasmatales archaeon	2.08649	0.01
mcrA	K00399	Thermoplasmatales archaeon	2.060044	0.01
mtbB	K16178	*Methanomethylophilus alvus*	2.037695	0.02
mcrA	K00399	*Methanomethylophilus alvus*	2.026371	0.01
mtmB	K16176	*Methanomethylophilus alvus*	2.013867	0.02
mcrB	K00401	Thermoplasmatales archaeon	1.97292	0.02
mcrA	K00399	*Methanomethylophilus alvus*	1.970599	0.02
mcrA	K00399	Thermoplasmatales archaeon	1.966109	0.02

mvhG	K14128	*Methanomethylophilus alvus*	1.95798	0.02
mcrA	K00399	*Methanomethylophilus alvus*	1.95451	0.01
mtmB	K16176	*Methanomethylophilus alvus*	1.941464	0.02
mcrA	K00399	Thermoplasmatales archaeon	1.901289	0.01
mtmB	K16176	*Methanomethylophilus alvus*	1.8886	0.01
hdrA2	K03388	Thermoplasmatales archaeon	1.880038	0.02
mcrA	K00399	Thermoplasmatales archaeon	1.86647	0.03
mcrB	K00401	*Methanomethylophilus alvus*	1.864139	0.03
mcrG	K00402	Thermoplasmatales archaeon	1.8376	0.02
hdrA2	K03388	Thermoplasmatales archaeon	1.829351	0.04
mtbB	K16178	*Methanomethylophilus alvus*	1.824865	0.04
mcrB	K00401	Thermoplasmatales archaeon	1.810528	0.03
mcrA	K00399	*Methanomethylophilus alvus*	1.807524	0.02
mtmB	K16176	*Methanomethylophilus alvus*	1.804866	0.03
mcrG	K00402	*Methanomethylophilus alvus*	1.801436	0.04
mcrB	K00401	Thermoplasmatales archaeon	1.770492	0.02
mcrA	K00399	*Methanomethylophilus alvus*	1.760893	0.02
hdrB2	K03389	*Methanomethylophilus alvus*	1.756497	0.04
mcrA	K00399	*Methanomethylophilus alvus*	1.752823	0.03
mcrB	K00401	Thermoplasmatales archaeon	1.746377	0.04
hdrD	K08264	*Methanomethylophilus alvus*	1.728693	0.03
mcrG	K00402	*Methanomethylophilus alvus*	1.727742	0.05
mcrA	K00399	Thermoplasmatales archaeon	1.726943	0.04
mcrA	K00399	*Methanomethylophilus alvus*	1.712247	0.04
mcrA	K00399	*Methanomethylophilus alvus*	1.67934	0.05
mcrA	K00399	*Methanomethylophilus alvus*	1.667273	0.04
hdrD	K08264	*Methanomethylophilus alvus*	1.656392	0.05
mcrA	K00399	*Methanomethylophilus alvus*	1.407736	0.05
Involved in KEGG Module M00567				
mcrB	K00401	Thermoplasmatales archaeon	4.28647	< 0.01
mcrA	K00399	Thermoplasmatales archaeon	3.080039	< 0.01
mcrB	K00401	Thermoplasmatales archaeon	3.015968	< 0.01
mcrA	K00399	*Methanomethylophilus alvus*	2.867172	< 0.01
hdrC2	K03390	*Methanomethylophilus alvus*	2.839452	< 0.01
mcrB	K00401	Thermoplasmatales archaeon	2.751106	< 0.01
hdrD	K08264	*Methanomethylophilus alvus*	2.416595	< 0.01
mcrA	K00399	*Methanomethylophilus alvus*	2.38888	< 0.01
mcrA	K00399	Thermoplasmatales archaeon	2.233378	< 0.01
mcrA	K00399	*Methanomethylophilus alvus*	2.227565	< 0.01
mcrA	K00399	*Methanomethylophilus alvus*	2.201209	< 0.01
mcrA	K00399	*Methanomethylophilus alvus*	2.180225	< 0.01
mcrA	K00399	*Methanomethylophilus alvus*	2.116668	0.01
mcrB	K00401	Thermoplasmatales archaeon	2.08649	0.01
mcrA	K00399	Thermoplasmatales archaeon	2.060044	0.01

mcrA	K00399	*Methanomethylophilus alvus*	2.026371	0.01
mcrB	K00401	Thermoplasmatales archaeon	1.97292	0.02
mcrA	K00399	*Methanomethylophilus alvus*	1.970599	0.02
mcrA	K00399	Thermoplasmatales archaeon	1.966109	0.02
mvhG	K14128	*Methanomethylophilus alvus*	1.95798	0.02
mcrA	K00399	*Methanomethylophilus alvus*	1.95451	0.01
mcrA	K00399	Thermoplasmatales archaeon	1.901289	0.01
hdrA2	K03388	Thermoplasmatales archaeon	1.880038	0.02
mcrA	K00399	Thermoplasmatales archaeon	1.86647	0.03
mcrB	K00401	*Methanomethylophilus alvus*	1.864139	0.03
mcrG	K00402	Thermoplasmatales archaeon	1.8376	0.02
hdrA2	K03388	Thermoplasmatales archaeon	1.829351	0.04
mcrB	K00401	Thermoplasmatales archaeon	1.810528	0.03
mcrA	K00399	*Methanomethylophilus alvus*	1.807524	0.02
mcrG	K00402	*Methanomethylophilus alvus*	1.801436	0.04
mcrB	K00401	Thermoplasmatales archaeon	1.770492	0.02
mcrA	K00399	*Methanomethylophilus alvus*	1.760893	0.02
hdrB2	K03389	*Methanomethylophilus alvus*	1.756497	0.04
mcrA	K00399	*Methanomethylophilus alvus*	1.752823	0.03
mcrB	K00401	Thermoplasmatales archaeon	1.746377	0.04
hdrD	K08264	*Methanomethylophilus alvus*	1.728693	0.03
mcrG	K00402	*Methanomethylophilus alvus*	1.727742	0.05
mcrA	K00399	Thermoplasmatales archaeon	1.726943	0.04
mcrA	K00399	*Methanomethylophilus alvus*	1.712247	0.04
mcrA	K00399	*Methanomethylophilus alvus*	1.67934	0.05
mcrA	K00399	*Methanomethylophilus alvus*	1.667273	0.04
hdrD	K08264	*Methanomethylophilus alvus*	1.656392	0.05
mcrA	K00399	*Methanomethylophilus alvus*	1.407736	0.05
Archaeal DEGs downregulated at H4				
Involved in KEGG Module M00356				
mcrA	K00399	*Methanobrevibacter ruminantium*	-2.805356	< 0.01
hdrA2	K03388	*Methanobrevibacter smithii*	-2.439509	< 0.01
mcrA	K00399	*Methanobrevibacter smithii*	-2.422385	< 0.01
mtaA	K14080	Thermoplasmatales archaeon	-2.401648	< 0.01
hdrA2	K03388	Thermoplasmatales archaeon	-2.399049	< 0.01
hdrD	K08264	*Methanosarcina barkeri*	-2.306421	< 0.01
hdrA2	K03388	*Methanobrevibacter smithii*	-2.303998	< 0.01
mcrG	K00402	*Methanobrevibacter smithii*	-2.277285	< 0.01
hdrA2	K03388	*Methanobrevibacter smithii*	-2.125172	0.01
mcrA	K00399	*Methanobrevibacter smithii*	-2.106201	0.01
fdhB	K00125	*Methanobrevibacter ruminantium*	-2.100255	0.01
hdrA2	K03388	Thermoplasmatales archaeon	-2.07224	0.01
hdrA2	K03388	*Methanobrevibacter smithii*	-2.047941	0.01
mcrB	K00401	*Methanobrevibacter smithii*	-1.938398	0.03

mcrB	K00401	*Methanobrevibacter smithii*	-1.88637	0.03
hdrA2	K03388	*Methanobrevibacter smithii*	-1.861374	0.02
hdrA2	K03388	*Methanomethylophilus alvus*	-1.856806	0.03
mvhG	K14128	*Methanomethylophilus alvus*	-1.830586	0.03
hdrA2	K03388	*Methanobrevibacter smithii*	-1.808439	0.04
mtaB	K04480	*Methanomethylophilus alvus*	-1.763466	0.04
fdhB	K00125	*Methanobrevibacter smithii*	-1.762956	0.01
Involved in KEGG Module M00357				
mtrH	K00584	*Methanobrevibacter smithii*	-3.170991	< 0.01
mtrA	K00577	*Methanobrevibacter smithii*	-3.146641	< 0.01
mtrA	K00577	*Methanobrevibacter smithii*	-3.11275	< 0.01
mcrA	K00399	*Methanobrevibacter ruminantium*	-2.805356	< 0.01
mtrH	K00584	*Methanobrevibacter smithii*	-2.721475	< 0.01
mtrH	K00584	*Methanobrevibacter smithii*	-2.598171	< 0.01
hdrA2	K03388	*Methanobrevibacter smithii*	-2.439509	< 0.01
mcrA	K00399	*Methanobrevibacter smithii*	-2.422385	< 0.01
hdrA2	K03388	Thermoplasmatales archaeon	-2.399049	< 0.01
acs	K01895	*Methanomicrobium mobile*	-2.343782	< 0.01
hdrD	K08264	*Methanosarcina barkeri*	-2.306421	0.01
hdrA2	K03388	*Methanobrevibacter smithii*	-2.303998	0.01
mcrG	K00402	*Methanobrevibacter smithii*	-2.277285	0.01
acs	K01895	*Methanobrevibacter smithii*	-2.260681	0.01
mtrD	K00580	*Methanobrevibacter smithii*	-2.226262	0.01
hdrA2	K03388	*Methanobrevibacter smithii*	-2.125172	0.01
mcrA	K00399	*Methanobrevibacter smithii*	-2.106201	0.01
fdhB	K00125	*Methanobrevibacter ruminantium*	-2.100255	0.01
hdrA2	K03388	Thermoplasmatales archaeon	-2.07224	0.01
hdrA2	K03388	*Methanobrevibacter smithii*	-2.047941	0.01
mcrB	K00401	*Methanobrevibacter smithii*	-1.938398	0.03
mcrB	K00401	*Methanobrevibacter smithii*	-1.88637	0.03
hdrA2	K03388	*Methanobrevibacter smithii*	-1.861374	0.02
hdrA2	K03388	*Methanomethylophilus alvus*	-1.856806	0.03
mvhG	K14128	*Methanomethylophilus alvus*	-1.830586	0.03
hdrA2	K03388	*Methanobrevibacter smithii*	-1.808439	0.04
mtrH	K00584	*Methanobrevibacter ruminantium*	-1.778831	0.05
fdhB	K00125	*Methanobrevibacter smithii*	-1.762956	0.01
mtrH	K00584	*Methanobacterium arcticum*	-1.6837	0.05
mtrA	K00577	*Methanobrevibacter smithii*	-1.682932	0.05
Involved in KEGG Module M00563				
mcrA	K00399	*Methanobrevibacter ruminantium*	-2.805356	< 0.01
mttB	K14083	Thermoplasmatales archaeon	-2.702456	< 0.01
hdrA2	K03388	*Methanobrevibacter smithii*	-2.439509	< 0.01
mcrA	K00399	*Methanobrevibacter smithii*	-2.422385	< 0.01
hdrA2	K03388	Thermoplasmatales archaeon	-2.399049	< 0.01

hdrD	K08264	*Methanosarcina barkeri*	-2.306421	0.01
hdrA2	K03388	*Methanobrevibacter smithii*	-2.303998	0.01
mcrG	K00402	*Methanobrevibacter smithii*	-2.277285	0.01
mttC	K14084	*Methanomethylophilus alvus*	-2.226272	0.01
mttB	K14083	*Methanomethylophilus alvus*	-2.191156	0.01
hdrA2	K03388	*Methanobrevibacter smithii*	-2.125172	0.01
mtbB	K16178	*Methanomethylophilus alvus*	-2.107874	0.01
mcrA	K00399	*Methanobrevibacter smithii*	-2.106201	0.01
fdhB	K00125	*Methanobrevibacter ruminantium*	-2.100255	0.01
hdrA2	K03388	Thermoplasmatales archaeon	-2.07224	0.01
mtmB	K16176	Thermoplasmatales archaeon	-2.055972	0.02
hdrA2	K03388	*Methanobrevibacter smithii*	-2.047941	0.01
mcrB	K00401	*Methanobrevibacter smithii*	-1.938398	0.03
mcrB	K00401	*Methanobrevibacter smithii*	-1.88637	0.03
hdrA2	K03388	*Methanobrevibacter smithii*	-1.861374	0.02
hdrA2	K03388	*Methanomethylophilus alvus*	-1.856806	0.03
mvhG	K14128	*Methanomethylophilus alvus*	-1.830586	0.03
hdrA2	K03388	*Methanobrevibacter smithii*	-1.808439	0.04
fdhB	K00125	*Methanobrevibacter smithii*	-1.762956	0.01
Involved in KEGG Module M00567				
mtrH	K00584	*Methanobrevibacter smithii*	-3.170991	< 0.01
mtrA	K00577	*Methanobrevibacter smithii*	-3.146641	< 0.01
mtrA	K00577	*Methanobrevibacter smithii*	-3.11275	< 0.01
mcrA	K00399	*Methanobrevibacter ruminantium*	-2.805356	< 0.01
mtrH	K00584	*Methanobrevibacter smithii*	-2.721475	< 0.01
mtrH	K00584	*Methanobrevibacter smithii*	-2.598171	< 0.01
hdrA2	K03388	*Methanobrevibacter smithii*	-2.439509	< 0.01
ftr	K00672	*Methanobrevibacter smithii*	-2.434585	< 0.01
fwdA	K00200	*Methanobrevibacter smithii*	-2.42532	< 0.01
mcrA	K00399	*Methanobrevibacter smithii*	-2.422385	< 0.01
hdrA2	K03388	Thermoplasmatales archaeon	-2.399049	< 0.01
hdrD	K08264	*Methanosarcina barkeri*	-2.306421	0.01
hdrA2	K03388	*Methanobrevibacter smithii*	-2.303998	0.01
mcrG	K00402	*Methanobrevibacter smithii*	-2.277285	0.01
mtrD	K00580	*Methanobrevibacter smithii*	-2.226262	0.01
fwdD	K00203	*Methanobrevibacter smithii*	-2.206789	0.01
fwdA	K00200	*Methanobrevibacter smithii*	-2.135044	0.01
hdrA2	K03388	*Methanobrevibacter smithii*	-2.125172	0.01
mcrA	K00399	*Methanobrevibacter smithii*	-2.106201	0.01
fdhB	K00125	*Methanobrevibacter ruminantium*	-2.100255	0.01
hdrA2	K03388	Thermoplasmatales archaeon	-2.07224	0.01
hdrA2	K03388	*Methanobrevibacter smithii*	-2.047941	0.01
fwdA	K00200	*Methanobrevibacter smithii*	-1.974988	0.02
mcrB	K00401	*Methanobrevibacter smithii*	-1.938398	0.03

mer	K00320	*Methanobrevibacter ruminantium*	-1.906796	0.01
mcrB	K00401	*Methanobrevibacter smithii*	-1.88637	0.03
hmd	K13942	*Methanobrevibacter ruminantium*	-1.873352	0.03
hdrA2	K03388	*Methanobrevibacter smithii*	-1.861374	0.02
hdrA2	K03388	*Methanomethylophilus alvus*	-1.856806	0.03
mvhG	K14128	*Methanomethylophilus alvus*	-1.830586	0.03
hdrA2	K03388	*Methanobrevibacter smithii*	-1.808439	0.04
fwdA	K00200	*Methanobrevibacter smithii*	-1.799232	0.04
mtrH	K00584	*Methanobrevibacter ruminantium*	-1.778831	0.05
fdhB	K00125	*Methanobrevibacter smithii*	-1.762956	0.01
mtrH	K00584	*Methanobacterium arcticum*	-1.6837	0.05
mtrA	K00577	*Methanobrevibacter smithii*	-1.682932	0.05
