# Supplementary material for: Ruminal archaea and bacteria metatranscriptomic responses to supplementation in steers fed low-quality forage
Source: J Anim Sci. 2026 Jun 12;104:skag188. doi: 10.1093/jas/skag188 (PMC13317134; doi:10.1093/jas/skag188)
Supplement: skag188_Supplementary_Data [file skag188_supplementary_data.zip › Supplemental_Table_1.pdf]

Supplemental Table 1. Summary of RNA sequencing read processing, quality metrics, and taxonomic filtering

| **Sample** | **RIN Value** | **# of Raw Read Pairs (M)** | **# of Trimmed Read Pairs (% of raw)** | **# of rRNA filtered Read Pairs* (% of raw)** | **rRNA detected (% of trimmed)** | **Archaeal Read Pairs** (% of trimmed & rRNA filtered)** |
|---|---|---|---|---|---|---|
| 23108Ark_12-S1 | 10 | 277 | 211 (86%) | 182 (66%) | 14% | 12.9 (7%) |
| 23108Ark_13-S2 | 8.5 | 222 | 186 (84%) | 157 (71%) | 16% | 10.0 (6.3%) |
| 23108Ark_14-S3 | 10 | 267 | 224 (84%) | 189 (71%) | 16% | 10.4 (5.4%) |
| 23108Ark_15-S4 | 10 | 197 | 126 (69%) | 108 (55%) | 14% | 5.1 (5.0%) |
| 23108Ark_16-S5 | 10 | 263 | 221 (84%) | 176 (67%) | 20% | 9.7 (5.4%) |
| 23108Ark_17-S6 | 10 | 216 | 184 (85%) | 152 (70%) | 17% | 7.3 (4.7%) |
| 23108Ark_18-S7 | 10 | 201 | 165 (82%) | 133 (66%) | 19% | 7.2 (5.3%) |
| 23108Ark_19-S8 | 8.1 | 232 | 197 (85%) | 163 (70%) | 17% | 7.1 (4.3%) |
| 23108Ark_20-S9 | 7.7 | 188 | 158 (84%) | 118 (63%) | 25% | 9.2 (7.7%) |
| 23108Ark_41-S16 | 4.8 | 223 | 187 (84%) | 166 (74%) | 11% | 11.4 (6.8%) |
| 23108Ark_43-S17 | 8.2 | 208 | 168 (81%) | 157 (75%) | 7% | 16 (10.10%) |
| 23108Ark_44-S18 | 7.7 | 169 | 145 (86%) | 129 (76%) | 11% | 10.7 (8.3%) |
| 23108Ark_45-S19 | 10 | 210 | 183 (87%) | 145 (69%) | 20% | 9.5 (6.4%) |
| 23108Ark_46-S20 | 8.8 | 134 | 117 (87%) | 89 (64%) | 24% | 6.1 (6.7%) |
| 23108Ark_47-S21 | 10 | 133 | 109 (82%) | 99 (74%) | 9% | 7.3 (7.3%) |
| 23108Ark_48-S22 | 10 | 149 | 130 (87%) | 117 (79%) | 10% | 8.7 (7.4%) |
| 23108Ark_49-S23 | 7.5 | 172 | 151 (88%) | 133 (77%) | 12% | 6.3 (4.7%) |
| 23108Ark_50-S24 | 10 | 147 | 122 (83%) | 109 (74%) | 11% | 6.0 (5.4%) |
| Range | | 133-277 | 109-224 (69-88%) | 89-189 (45-79%) | 7-25% | 5.1-12.9 (4.3-10.1%) |
| Average | | 200 | 166 (84%) | 140 (70%) | 15% | 8.9 (6%) |

* Trimmed read pairs were first run through bowtie2 to remove host contamination, which accounted for <2% of reads in all samples.

** Read pairs remaining after taxonomy filtering using kraken2 to remove bacterial, viral, protozoan, and fungal reads.
